# Comparative Study of the Use of Insect Meal from *Spodoptera littoralis* and *Bactrocera zonata* for Feeding Japanese Quail Chicks

**DOI:** 10.3390/ani9040136

**Published:** 2019-03-31

**Authors:** Waheed A. A. Sayed, Nashaat S. Ibrahim, Mahmoud H. Hatab, Fen Zhu, Birgit A. Rumpold

**Affiliations:** 1 Biological Application Department, Nuclear Research Center, Atomic Energy Authority, 3 ahmed elZomer St, Naser city, Cairo 11787, Egypt; waheed.sayed@eaea.org.eg (W.A.A.S.); nashaat1977@yahoo.com (N.S.I.); hatabmahmoud@yahoo.com (M.H.H.); 2 Hubei Insect Resources Utilization and Sustainable Pest Management Key Laboratory, College of Plant Science and Technology, Huazhong Agricultural University, Hongshan District, Wuhan 430070, China; zhufen@mail.hzau.edu.cn; 3 Department Education for Sustainable Nutrition and Food Science, Technische Universität Berlin, Marchstr. 23, D-10585 Berlin, Germany

**Keywords:** biochemical analysis, edible insects, feed insects, insect meals, feeding trial, poultry feed, soy replacement, sustainable feed

## Abstract

**Simple Summary:**

With the increasing world population and rising meat consumption, sustainable high-quality feed is urgently needed. It was proposed that insects could be a promising alternative feed source, since they have high protein contents and can be reared sustainably on food production residues. Caterpillars of the African cotton leafworm *Spodoptera littoralis* can be cultivated on different residues of crops and vegetables and larvae of the peach fruit fly *Bactrocera zonata* can be reared on fruit and vegetable residues. In addition, mass rearing expertise of these two insect species already exists thanks to the sterile insect technique, a method of biological pest control. The nutrient composition of meals from these two insect species was determined and the impact of their meals as feed ingredients for Japanese quail chicks in comparison to soybean meal was investigated. In the feeding trials, 50% of the soybean protein was replaced with *B. zonata* meal and *S. littoralis* meal, respectively. For chicks fed with insect meal containing diets, improved growth, feed performance parameters, carcass characteristics and biochemical indices were observed in comparison to a soybean meal-based diet. Consequently, both insect meals represent a promising alternative to soy in the feed of Japanese quail chicks.

**Abstract:**

A transformation of current livestock production towards a more sustainable operation is crucial to face nutritional and environmental challenges. There is an urgent demand for more sustainable high-quality feed sources to reduce environmental costs. Insects pose a potential alternative since they can be reared sustainably on food and feed residues. Know-how in mass rearing already exists for insect species used in biological pest control, such as the African cotton leafworm *Spodoptera littoralis* and the peach fruit fly *Bactrocera zonata*. The impact of a replacement of 50% of soybean meal by *S. littoralis* and *B. zonata* meal, respectively, on seven-days-old Japanese quail chicks was investigated in feeding trials. Concomitantly, the chemical compositions of the two insect meals and soybean meal were determined and compared. It was observed that the insect meals had higher protein and fat contents, lower carbohydrate contents and contained more saturated fatty acids than soybean meal. They also had higher methionine, and *S. littoralis* had a higher lysine content. Feeding trials resulted in improved growth, feed performance parameters, carcass characteristics, and biochemical indices for both insect meals. Consequently, both insect meals represent a promising alternative to soy in the feed of Japanese quail chicks.

## 1. Introduction

Since feed was identified as one of the major contributors to land occupation, primary production use, acidification, climate change, energy use and water dependence [1], there is an urgent demand for more sustainable high-quality feed sources to reduce the environmental costs of aquaculture and livestock production. A transformation of current livestock production towards a more sustainable operation is crucial to face the nutritional challenge as well as meet the sustainable development goals (SDGs) of the UN.

With that being said, livestock production generally requires high-quality protein as feed [2]. Soybean, fish meal and animal protein meal are commonly used as protein sources in poultry feed in Egypt. The use of meat protein meals has added constraint to poultry production due to its involvement in the transmission of diseases. Nevertheless, meat is still used in a number of developing countries [3], and therefore it is necessary to search for locally available, sustainable, safe and low-cost feedstuffs to replace fish meal and other traditional animal protein sources as well as soybean. Edible insects have been suggested as a potential sustainable alternative source for livestock feed because of their high-value protein, their beneficial nutrient composition and their comparably low environmental impact [4]. It is proposed that edible insects can play an important role as a sustainable feed source since they are able to transform and valorize low nutritive products into high-quality feed with limited pressure on global land, water and energy resources. Lepidopteran and Dipteran insects in particular are suggested as an alternative protein source for livestock feed due to their comparably high contents in high quality protein, and also because they can be produced in a short amount of time and can reared at low costs on different side stream-residues [5,6]. It has been reported that a number of edible insects could be applied in livestock feed. For example, insect species such as the yellow mealworm *Tenebrio molitor* (Coleoptera) and black soldier fly *Hermetia illucens* (Diptera) are applicable as food and feed [7]. They are successfully used in aquaculture [8,9,10,11,12,13], and also as an alternative source of protein in poultry feed [10,14,15,16].

The African or Egyptian cotton leafworm *Spodoptera littoralis* is a common caterpillar (Lepidoptera: Noctuidae) in Africa and feeds on many different crops and vegetables, while the peach fruit fly *Bactrocera zonata* (Diptera: Tephritidae) feeds on a wide range of fruits and vegetables [17]. In this context, these two species could be processed sustainably into feedstuffs using food residues. In general, the late larval stages in Lepidopteran and Dipteran insects are particularly recommended to utilize as feedstuff, since they are easy to collect and harvest, large in size and have high nutrient contents [18,19,20,21,22]. A major advantage of using insects like *B. zonata* as feed insects is the fact that rearing expertise and mass rearing facilities already exist due to existing insect production for biological insect pest control using sterile insect technique (SIT) in. In the present study, two different insect meals derived from *B. zonata* and *S. littoralis* larvae were compared in Japanese quail diets. The impact of insect-based feed on the productive performance (growth performance and carcass characteristics) and animal welfare (evaluated by the blood traits) of Japanese quail was investigated.

## 2. Materials and Methods 

### 2.1. Animal Ethics

All of the experiments were carried out according to the guidelines of the Institutional Animal Care and Use Committee (IACUC) for animal experiments, which is a member in the Egyptian Network of Research Ethics Committee (ENERC). The scientific and ethics committee of the Biological Application Department, Nuclear Research Center, Egypt, approved all procedures used in this experiment (protocol number 176; date of approval: 23-5-2018).

### 2.2. Insect Rearing and Harvest

Both the Cotton leafworm *S. littoralis* and the peach fruit fly *B. zonata* were reared and maintained in the insect farm of the Department of Biological Application, Nuclear Research Center (NRC), Egyptian Atomic Energy Authority (EAEA). The adults of *S. littoralis* were fed on sucrose solution (10% *w*/*v)* while the adult diet of *B. zonata* consisted of one part yeast hydrolyzate (enzymatic) and three parts sucrose. The rearing cages of the flies were additionally provided with drinking water. The hatched larvae of *S. littoralis* were reared on castor bean *Ricinus communis* leaves until the last instar (sixth instar) within 13–15 days, while the larvae of *B. zonata* were reared on a semi-artificial diet consisting of 28% wheat bran, 7% yeast, 3% sucrose, 0.28% sodium benzoate, 1.5% HCl and 50.20% water until the last instar (third instar) within seven to eight days. The rearing conditions were 25 ± 2 °C, 60 ± 5% relative humidity (RH), with a photoperiod of 14 L: 10 D hours. The full grown larvae were collected, ground using a blender (3.8 liter) (Hamiliton Beach blender model HBF900S, VA, USA) and then frozen for storage. Frozen, ground larvae of *S. littoralis* and *B. zonata* were oven-dried at 40 °C for 24 h before use in feeding trials and analyses of their chemical compositions were performed.

### 2.3. Chemical Analyses

The amino acid profiles of *B. zonata*, *S. littoralis* and soybean meal were analyzed by high-performance liquid chromatography (HPLC) following the method by Alikwe et al. [23]. Moreover, carbohydrate content was estimated following the method according to Albalasmeh [24]. The total lipids were extracted and purified according to Folch et al. [25]. The crude fat contents of the insect meals were analyzed according to Association of Official Analytical Chemists (AOAC) [26]. Fatty acid compositions of total lipids were analyzed as described by Knapp [27]. A gas chromatograph (hp 6890, Agilent, CA, USA) equipped with innowax-crosslinked polyethylene glycol column 30 m was used for deferential fatty acid analysis. Furthermore, elemental compositions were analyzed by an Energy Dispersive X-ray analyzer (EDX, Jeol, Japan) attached to a scanning electron microscope model HEOL-JSM 4500 (Jeol, Japan). All analyses were performed in triplicates, respectively.

### 2.4. Feeding Trials

The impact of replacing 50% of the soy bean meal with *B. zonata* meal and *S. littoralis* meal at a constant protein content on the performance of Japanese quails was investigated in feeding trials in comparison to the control diet. The chicks were maintained at the poultry experimental farm of the Biological Application Department, Nuclear Research Center, EAEA. A total of 360 Japanese quail chicks (180 male, 180 female), seven days old and weighing 22 g on average were randomly allotted three dietary treatments (120 chicks in each group), each consisting of three pens as replicates with 40 chicks (20 female and 20 male) per pen. Three basal diets based on corn–soybean meal were formulated and fed to the Japanese quail experimental groups. Three different sources of protein meal were used: soybean meal was used in the control diet, 50% of the soybean meal was substituted with *B. zonata* meal in diet A and 50% of the soybean protein was substituted with *S. littoralis* meal in diet B (Table 1). All diets were formulated to meet the nutrient requirements of Japanese quail chicks [28]. In order to maintain roughly the same protein content of 22% in all three diets as recommended in the literature [28,29], additional corn meal and oil were added to the insect diets (A and B). This, as well as the replacement of soybean meal, resulted in ~11% higher energy contents (approx.. 300 kcal/kg) of the insect-containing diets in comparison to the control diet. The three feeding trials were carried out in 45 days. All groups were farmed in electrically heat-controlled batteries: in the first seven days the temperature was controlled at 35 °C, while 28 ± 2 °C was maintained after that until the end of the experiment. RH was 50 ± 5% and photoperiod was 14 L: 10 D hours.

### 2.5. Growth Performance

Body weight gain, feed intake and feed conversion ratio were determined for the overall experimental period (38 days). All measurements were performed on the pen basis using a high precision electronic scale. Moreover, the mortality was monitored daily during the whole experimental period. Furthermore, at the end of an experimental period of 45 days, 10 quails from each feeding group (chosen on the basis of average pen final body weight, five females and five males) per pen were weighed and slaughtered for carcass analysis. Carcass, liver, heart and spleen weights were calculated for each slaughtered bird as well as a relative percentage of live body weight.

### 2.6. Biochemical and Hormones Analysis

Blood samples were collected from slaughtered birds and left to clot, then centrifuged at 4000 rpm for 10 min and the resulting serum was stocked at −20 °C for hormonal and chemical analyses. Serum total proteins, total antioxidant, triglyceride, total cholesterol, albumin, alanine aminotransferase (ALT), aspartate aminotransferase (AST) and alkaline phosphates (ALP) were determined using commercial kits produced by Stanbio Company, USA using a computerized spectrophotometer (model Milton Roy 1201, Ivyland Road Ivyland, PA, USA). Serum globulin values were calculated by subtracting albumin values from their corresponding total protein values of the same sample. All analyses were performed in triplicates, respectively.

### 2.7. Statistical Analysis 

One-way analysis of variance was done using SAS software version 9.1.3 (Sas institute, NC, USA) following the General Linear Model procedure with dietary treatment as fixed effect. Mean values were assessed for significance using multiple range tests [30]. The data on the relative weight of carcass and some organs were arcsine transformed, whereas reported means and standard errors (SEs) were derived from the original data.

## 3. Results and Discussion

### 3.1. Nutritional Values of B. zonata and S. littoralis

Previous studies on the chemical composition of a variety of insect species showed their high nutritive value [31]. For instance, crude protein contents up to 40%–70% (on a dry matter (DM) basis), lipid contents as high as 9%–25% DM, unsaturated to saturated fatty acid ratio up to 5.8–8.2 and crude fiber contents of 0.93%–11% DM were found [19,31,32,33]. The present study showed that the nutritional compositions of *B. zonata*, *S. littoralis* and soybean meals differed (Table 2, Table 3 and Table 4). The crude protein content of the *B. zonata* meal (58.1% DM) was slightly higher than of the *S. littoralis* meal (51.2% DM) and soybean meal (44.0% DM) as shown in Table 2. In general, the protein content varied for each insect species from 13% to 70% [34,35], while Rumpold and Schlüter [31] reported that the average protein contents of insects ranged from 35.34% for the order Isoptera (termites) to 61.32% for the order Orthoptera (crickets, grasshoppers, locusts). It can be concluded that the protein content of both insect species investigated is high, with values above 50% (based on dry matter (DM)). The crude lipid content of the *S. littoralis* meal (33.1%) was higher than of the *B. zonata* meal (25.3%), and considerably higher than the soybean meal (1.9%). The carbohydrate and fiber contents of the *B. zonata* and *S. littoralis* meals were comparable with 4.9% and 5.2% carbohydrates and 10.9% and 10.7% fibers, respectively, while the carbohydrate content of the soybean meal was much higher (28.51%), and its fiber content was slightly lower at 7.3%. 

It can be concluded that both insect meals contain sufficient amounts of protein in comparison to soy bean meal. The considerably higher lipid contents of both insect meals could require a defattening step of the meals before their use as livestock feed. However, it could also result in a reduction of the addition of supplementary lipids such as corn oil.

### 3.2. Amino Acid Content

In Table 3, the essential and non-essential amino acid contents of both insect meals and the soybean meal are presented. Roughly similar amino acid spectra were obtained for both insect meals as compared to the soybean meal. Remarkable are the higher methionine contents in the meals of both insect species, and the higher lysine content in the *S. littoralis* meal than in the soybean meal. In diets for Japanese quails and other poultry that are based on corn–soybean meal, methionine is a growth-limiting amino acid that is supplemented [36]. Therefore, the higher methionine content of insect meals could result in a decreased supplementation with synthetic DL-methionine.

Furthermore, contents of the essential amino acids proline and histidine were higher in *B. zonata* than in the *S. littoralis* and soybean meals, while the leucine content was higher in *S. littoralis* than in the *B. zonata* and soybean meals. The contents of the non-essential amino acids arginine and glutamic acid were higher in the soybean meal than the two insect meals, while tyrosine and arginine were somewhat higher in the *B. zonata* than in the *S. littoralis* meal. The obtained data are coincident with DeFoliart [37] and Makkar [19], who stated that the amino acids methionine and cysteine are low in insect proteins. The obtained data revealed that the two tested insects were rich in the amino acids aspartic acid, lysine and proline. This is only partly in accordance with scientific literature where it was reported that phenylalanine, tyrosine, leucine and lysine contents are the highest in Lepidopteran and Dipteran species [31,34,38].

Due to an additional supplementation with lysine and methionine of 0.1% (1 g/kg) in all three experimental diets (Table 1), respectively, no definite conclusion on the impact of the differing amino acid compositions on growth performance of quail chicks can be drawn. However, since the lysine and methionine requirements of Japanese quail chicks are 1.3% and 0.5% [28], respectively, these are significantly higher than the 0.1% supplied by the vit & min premix (see Table 1), and the protein source has been determined to have an impact.

### 3.3. Lipid and Fatty Acid Contents

According to van Huis et al. [5], insects are a considerable source of fat. This was also true for the two insects species investigated in this study. The obtained data in Table 2 shows that the crude lipid contents of *S. littoralis* (25.3%) and *B. zonata* (33.1%) were not only higher than those detected in the soybean meal (1.9%), as mentioned before, but also higher than values reported for some other species in the insect order Lepidoptera such as *Anaphe recticulata* (10%), *Bombyx mori* (9.5%), *Polyrhachis vicina* (6.0%) [6,39] and for the Dipteran species *Drosophila melanogaster* (17.9%) and *Eristalis species* (11.8%) [40,41]. However, they were lower than lipid contents reported by other authors [41,42,43] for Lepidopteran insects such as *Galleria mellonella* (60.0%), *Xyleutes redtembacheri* (48.0%) and *Comadia redtembacheri* (47.9%) , respectively, and some Dipteran insects such as *Copestylum anna* (31.0%) and *Ephydra hians* (41.0%) [41,43]. Moreover they were comparable to lipid contents obtained by Raksakantong et al. [44] for *Bombyx mori* (30.0), by Sirimungkararat et al. [43] for *Heliothis zea* (29.0%) (both of the order Lepidoptera) and by Ekpo et al. [45] for *Musca domestica* (24%) (Diptera).

Since the lipid content of soybean meal is comparably low and negligible in comparison, its impact as a lipid and fatty acid source is also low. Therefore, the fatty acid composition of corn oil was considered as well. As shown in Table 4, both insect meals contained both saturated and unsaturated fatty acids. Overall, the saturated fatty acid contents of both insect meals were 3.5 times higher than of the soybean meal and corn oil, whereas the unsaturated fatty acid contents were lower in comparison. The comparatively high amount of saturated fatty acids is typical for animal-derived fat and is in accordance with Rumpold and Schlüter [31], who reviewed the average amounts of saturated fatty acids of insect orders from 30% for Hymenoptera to 41% for Isoptera, and unsaturated fatty acids from 15% for Diptera to 39.76% for Lepidoptera and 48% for Hymenoptera. In comparison, both the Lepidopteran *B. zonata* and the Diperan *S. littoralis* in our insect meal samples had high saturated fatty acid contents of 41.45% and 46.18%, respectively. The palmitoleic and oleic acid contents in the *S. littoralis* meal were higher than in the *B. zonata* meal, while the palmitic, tricosylic and lignoceric acid contents were somewhat similar in the two insect meals, and absent in the soybean meal. The detection of tricosylic acid in the insect oils was remarkable, since fatty acids with an uneven number of carbon atoms are seldomly found in nature. Moreover, the linoleic acid content was more than two times higher in the soybean meal and 3.5–4 times higher in corn oil than in the two insect meals. Regarding the much higher lipid content of both insect meals, they did actually contain more linoleic acid than the soybean meal. The *B. zonata* meal contained 3.92% and the *S. littoralis* meal 4.51% linoleic acid, whereas the soybean meal contained only 0.76% linoleic acid. Lipid requirements of Japanese quail feed comprise 1% linoleic acid at the growing stage [28], but because corn oil was added as supplementary lipid source in all diets, no conclusions can be drawn on the contribution of the insect meals to the quails´ lipid provisions. Theoretically, 1 kg of quail chick feed would have to contain 255 g *B. zonata* meal or 222 g *S. littoralis* meal in order to provide 10 g (1%) of linoleic acid without additional lipid sources. It can thus be concluded that the insect meal contents in diet A and B were both not high enough to be the sole provider of linoleic acid to meet the quails’ requirement. The corn oil supplement could be reduced, however. Furthermore, the data showed that linolenic, stearic and arachidic acids were not detected in the two insect meals, but could be found in the soybean meals. 

### 3.4. Trace Elements

The elements phosphorous (P), sulphur (S), potassium (K), calcium (Ca), iron (Fe), magnesium (Mg), chlorine (Cl) and zinc (Zn) were found in both the *B. zonata* and *S. littoralis* meals (Table 5). *B. zonata* also contained silicium (Si), whereas Si, Cl and S were not detected in the soybean meal.

The contents in Ca, Mg and P were higher in the *B. zonata* meal than in the *S. littoralis* and soybean meals. Generally, the Zn content of both insect species was lower, and the Ca content of both insect species was considerably higher than in the soybean meal. The Ca content is especially of interest since, for animal nutrition, Ca plays vital roles in many enzyme-mediated processes like blood clotting and bone building, as well as by virtue of its phosphate salts in neuromuscular function [47]. The K content of the *S. littoralis* meal was higher than that recorded for the *B. zonata* and soybean meals, while the Fe, Zn and Mg contents of *S. littoralis* were lower than in *B. zonata*. Furthermore, Mg and S contents were relatively similar in the meals of the two tested insect species. 

In literature reviews, a large variation was noticed in the elemental contents of insect orders and species. For example, the grasshopper *Locusta migratoria* contains 8–20 mg iron per 100 g of dry matter, while the caterpillar of the moth *Gonimbrasia belina* contains 31–77 mg per 100 g of dry matter [48,49]. This was also attributed to different feed compositions [31].

The elemental contents in insects could be used as indicators for contaminative environments, and might indicate that insects have resistance to respective heavy metals. Previous studies on the black solider fly indicated that it could uptake and accumulate Cd from contaminated substrates [20,50,51]. For animal nutrition, it is crucial to prevent accumulation of heavy metals of feed insects via feed or other rearing conditions.

In addition, it has to be considered that other ingredients in the experimental diets could contribute to the trace element contents. Examples are the yellow corn meal and the mineral and vitamin premix. It has to be considered that if the composition of the premix should be altered upon substitution of the soybean meal with the two investigated insect meals, respectively, the lower iron content of both insect meals could require a higher iron content in the premix in comparison to the soybean meal.

### 3.5. Fibers and Carbohydrates

The data presented in Table 2 show that the fiber and carbohydrate contents in the two tested insect species were fairly similar and overall lower than in the soybean meal. The fiber contents of both insect species were much higher than the carbohydrate contents. In comparison, carbohydrate content was higher and fiber content was lower in the soybean meal than in the two insect meals. The common form of insect fiber is insoluble chitin contained mainly in their exoskeletons [52]. The amount of fiber in seven different insects species varied from *Locusta migratoria* (27%) to *Gryllus assimilis* (11%) based on dry matter. Chitin could improve the immune response and was considered to possess antibacterial properties towards pathogenic bacteria as well as antiviral properties [53]. It was also shown that chitin could function as an antifungal agent [54]. Moreover, Khoushab and Yamabhai [55] noticed antibacterial properties of chitin effective against bacteria such as *Escherichia coli*, *Vibrio cholerae*, *Shigella dysenteriae* and *Bacteroides fragilis*. The antibacterial mechanism of chitin is based on induced interaction between the positively charged chitin molecules and the negatively charged surface of the bacteria, resulting in leakage due to the cell wall of bacteria [56]. Another mechanism includes inducing the accumulation of pathogenesis-related proteins and chitinases for the activation of defense mechanisms in the host organism [57]. Since chitin content was not analyzed in this study, it cannot be concluded that the fiber content and chitin content are one and the same. Although the presence of chitin is certain, it is assumed that other fibers are present in insects.

The aforementioned results indicate that both *B. zonata* and *S. littoralis* meals could be considered a valuable source of protein and lipids for feedstuffs, but not of fibers. Moreover, it was hypothesized that the health and (more precisely) the gut health of poultry could be improved via feed containing the fiber chitin. In addition, it was suggested that the addition of antibiotics could be reduced in poultry production due to chitin-containing poultry feed [54,57].

### 3.6. Impact of B. zonata and S. littoralis Meal on Growing Performance of Japanese Quail

Results obtained in the feeding trials can be found in Table 6 and Table 7. The feed conversion ratios of Japanese quail were significantly increased (*p* ≤ 0.05) for diets containing *B. zonata* and *S. littoralis* meals, respectively, as compared with the control diet containing only soybean meal (Table 6). The highest feed intake and lowest body weight gain were observed for the control diet as compared to the *B. zonata* and *S. littoralis* meals (*p* ≤ 0.05). Moreover, significant differences were observed between the *B. zonata* and *S. littoralis* meals. The final weight was highest and the feed conversion ratio lowest for the *B. zonata* meal. 

The reduced feed intake and increased feed conversion ratio could be due to the increased dietary energy levels of the insect-containing diets, as observed by others [58,59]. On the other hand, Gheisari et al. [60] observed that dietary energy levels did not influence the feed conversion ratios of Japanese quails. In our study, the higher dietary level was mostly due to a higher lipid content in the insect-based diets, and it was suggested that feeding on different values of high-fat diet had no impact on growth performance, carcass composition and hormonal level composition [61,62]. In our study, diet B (with the *S. littoralis* meal) had the highest lipid and caloric energy contents. Nevertheless, the body weight gain and final weight were higher, and the feed conversion ratio lower, for quails fed *B. zonata* meal. Consequently, more research is required on iso-caloric diets and diets with the same lipid contents containing *B. zonata* and *S. littoralis* meals. Furthermore, the chicks remained healthy throughout the study, and no mortality occurred during the experimental period. This response could be due to the nutritional content of insect meal, which is highly nutritive. These results coincide with those observed by other authors [15,32,63,64]. In contrast, other studies have indicated that there are no differences between feeding insect meals and conventional meals on the growth performance of poultry birds [63,65,66,67]. The percentage of insect feed addition and the replacement of other feed ingredients such as soybean or fish meal with regard to the respective control diet (as well as the individual bird species) has to be considered when comparing these different feeding trials.

The percentages of carcass in the quail birds that were fed on the two tested insect meals were significantly higher than the control (Table 6). The comparably high weight of carcass could be due to the fact that *B. zonata* and *S. littoralis* meals have a high content of protein, essential amino acid (Lysine, phenyl-alnine, threonine and isoleucine), fat and energy. Meanwhile, the percentages of liver, heart, spleen and bursa were slightly higher for both insect meals compared with the control diet. These findings in the lymphoid organs can be attributed to an increase in the immune system of growing quail chicks. These results coincide with those obtained by Bovera et al. [4] and Schiavone et al. [15], who showed the highest weight of carcass and lymphoid organs in broilers fed *Tenebrio molitor* larvae meal as a protein source. The obtained results suggest that an inclusion of *B. zonata* and *S. littoralis* larvae meals in quail diets as a replacement for soybean meal improves the feed conversion ratio and promotes the growth of quail chicks.

### 3.7. Impact of B. zonata and S. littoralis Meals on Certain Hormonal Levels \

As shown in Table 7, there was an increase in serum total protein by using *B. zonata* and *S. littoralis* meal as compared to the control diet. This increase was higher for *B. zonata* meal than for *S. littoralis*. Moreover, the ratio of albumin to globulin was higher in the control meal than in the *B. zonata* and *S. littoralis* meals. Since total protein, albumen and globulin ratios are generally influenced by total protein intake, total serum protein may be applied as an indirect measurement of dietary protein quality [23]. Interestingly, the albumin to globulin ratio was low in the two tested insect meals. Abnormal serum albumin content usually indicates an alteration of normal systemic protein utilization. In addition, a low albumin:globulin ratio indicates better disease resistance and immune response of birds [4]. This is in line with other studies of broilers fed on *H. illucens* [68], of laying hens on *H. illucens* [69] and of Barbary partridges on *T. molitor* [70]. 

As depicted in the same table, the alkaline phosphates (ALP), alanine aminotransferase (ALT) and aspartate aminotransferase (AST) activities in all experimental groups were relatively similar and in the normal range. These data of liver functions could be important in the diagnosis of diseases as well as in the investigation and thorough assessment of feed, drugs and extracts used in feeding trials. They suggest that the insect-containing diets do not negatively affect the liver function of the quails. 

The data presented in Table 7 also shows that the triglyceride and cholesterol thyroxin values of quails fed with the *S. littoralis* and *B. zonata* meals, respectively, were significantly higher than the control. These results were in accordance with Loponte et al. [68], who observed high values in the serum of triglycerides and cholesterol in the Barbary partridge fed on *T. molitor*, as well as Duwa et al. [71], who observed significant differences in the triglycerides and cholesterol levels in blood of rabbits that were fed on fish meal and maggot meal. Furthermore, it is suggested that higher triglyceride levels might be due to the higher lipid contents of the two insect-containing diets.

## 4. Conclusions

The comparative study of two different insect species, *B. zonata* (Diptera) and *S. littoralis* (Lepidoptera), as a soybean meal replacement in feed for Japanese quail chicks showed that even if the two insects have slightly different nutritional compositions, meals from both insects can successfully replace 50% of soybean meal in the diets of growing quail chicks at a constant protein content of 22%.The feeding trials with insect meals resulted in improved growth, feed performance parameters, carcass characteristics and biochemical indices with regard to the control group fed only soybean meal as a protein source. It is suggested that the increased caloric energy content of the experimental diets containing insect meal might at least partly be responsible for the increased performance of Japanese quails in the feeding trials.

More research is required on the potential to replace soybean meal further (or even completely) with insect meal in the diet of Japanese quail chicks. It was also suggested that a supplementation of synthetic methionine as well as corn oil could be reduced by the use of either *B. zonata* and *S. littoralis* meal.

Furthermore, it needs to be determined whether meals of *S. littoralis* and *B. zonata* reared on food waste have the same capability to replace soybean meal at comparable performances. The transferability of the results obtained on other poultry as well as insect species needs to be investigated.

## Figures and Tables

**Table 1:** Ingredients and composition of experimental diets.

Diet Compositions (g/kg)	Control Diet	Diet A with *Bactrocera zonata* Meal	Diet B with *Spodoptera littoralis* Meal
Yellow corn	530.0	560.0	540.0
Soybean meal	400.0	200.0	200.0
Insect meal	0.0	152.0	173.0
Calcium carbonate	28.0	28.0	28.0
Corn oil	18.0	36.0	35.0
Dicalcium phosphate	15.0	15.0	15.0
Sodium chloride	3.0	3.0	3.0
Min & Vit premix *	3.0	3.0	3.0
Choline chloride	1.0	1.0	1.0
L-Lysine	1.0	1.0	1.0
DL-Methionine	1.0	1.0	1.0
Analyzed composition (g/100 g)			
Crude protein	22.1	22.5	22.24
Crude fiber	2.82	4.17	4.09
Crude fat	2.34	6.25	7.95
Gross caloric content (kcal/kg)	2811.5	3111.6	3124.5
Gross caloric content (kJ/kg)	11,763.3	13,018.9	13,072.9

* Vitamin and mineral premix provided per kg of premix: vit A, 1200 IU; vitD 1100 IU; vit E, 12 mg; vit B12, 0.02 mg; vit B1, 1 mg; choline chloride, 0.16 mg; copper, 3 mg; iron, 30 mg; manganese, 40 mg; zinc, 45 mg; and selenium, 3 mg.

**Table 2:** Average nutrient compositions of soybean, *B. zonata* and *S. littoralis* meals in % (based on dry matter).

Nutrient	(%) Soybean Meal	(%) *B. zonata* Meal	(%) *S. littoralis* Meal
Dry matter	97 ± 0.23	96 ± 0.35	95 ± 0.24
Total protein	44.0 ± 1.5 ^a^	58.1 ± 0.4 ^b^	51.2 ± 0.3 ^c^
Total lipid	1.9 ± 0.02 ^a^	25.3 ± 0.08 ^b^	33.1 ± 0.09 ^c^
Carbohydrates	28.51 ± 0.83 ^a^	4.9 ± 0.02 ^b^	5.2 ± 0.07 ^b^
Fiber	7.3 ± 0.52 ^a^	10.9 ± 0.04 ^b^	10.7 ± 0.05 ^b^

Means designated with different letters in the same row are significantly different (*p* ≤ 0.05).

**Table 3:** Relative amino acid contents in soybean, *B. zonata* and *S. littoralis* meals in %.

Essential Amino Acids	(%)			Non-Essential Amino Acids	(%)		
	Soybean Meal	*B. zonata*	*S. littoralis*		Soybean Meal	*B. zonata*	*S. littoralis*
Leucine	3.66 ± 0.92 ^a^	2.75 ± 0.01 ^c^	2.85 ± 0.04 ^b^	Aspartic acid	2.13 ± 0.3 ^c^	3.64 ± 0.05 ^a^	3.33 ± 0.06 ^b^
Lysine	2.99 ± 0.03 ^a^	2.32 ± 0.05 ^c^	3.55 ± 0.03 ^b^	Arginine	3.49 ± 0.1 ^a^	2.42 ± 0.03 ^b^	1.89 ± 0.002 ^c^
Proline	2.12 ± 0.31 ^c^	3.72 ± 0.02 ^b^	2.30 ± 0.08 ^a^	Glutamic acid	7.51 ± 0.23 ^a^	5.49 ± 0.04 ^b^	5.64 ± 0.05 ^b^
Valine	2.24 ± 0.67 ^b^	2.09 ± 0.03 ^c^	2.37 ± 0.06 ^a^	Alanine	1.23 ± 0.1 ^c^	2.21 ± 0.08 ^b^	2.73 ± 0.06 ^a^
Phenylalanine	2.35 ± 0.30 ^a^	2.71 ± 0.04 ^a^	1.86 ± 0.01 ^b^	Tyrosine	1.05 ± 0.04 ^a^	2.28 ± 0.01 ^b^	1.67 ± 0.01 ^c^
Isoleucine	2.15 ± 0.78 ^a^	1.66 ± 0.02 ^b^	1.80 ± 0.09 ^b^	Glycine	0.56 ± 0.01 ^c^	1.72 ± 0.07 ^b^	1.95 ± 0.03 ^a^
Threonine	1.89 ± 0.23	1.57 ± 0.01	1.57 ± 0.03	Serine	1.4 ± 0.02 b	1.64 ± 0.05 ^a^	1.67 ± 0.009 ^a^
Methionine	0.6 ± 0.69 ^a^	1.14 ± 0.04 ^b^	1.18 ± 0.04 ^b^	Cysteine	0.66 ± 0.075 ^b^	0.5 ± 0.004 ^c^	0.88 ± 0.005 ^a^
Histidine	1.21 ± 0.02 ^a^	2.31 ± 0.03 ^b^	1.91 ± 0.08 ^c^				

Means designated with different letters in the same row are significantly different (*p* ≤ 0.05). The *p* values were recorded *p* = 0.0000 *** for all amino acid contents except isoleucine (*p* = 0.0002 ***) and serine (*p* = 0.0004 ***).

**Table 4:** Relative fatty acids contents in the lipids of soybean, *B. zonata* and *S. littoralis* meals as well as of soybean meal in comparison to fatty acid composition of corn oil in %.

Fatty Acids (%)	C:n	Soybean Meal	*Corn oil* [46]	*B. zonata* Meal	*S. littoralis* Meal
Palmitic acid	C16:0	9.0 ± 0.4 ^c^	8.99	11.35 ± 0.23 ^b^	14.20 ± 0.16 ^a^
Stearic acid	C18:0	3.0 ± 0.4 ^a^	1.72	0.0 ^b^	0.0 ^b^
Arachidic acid	C20:0	0.7 ± 0.03 ^a^	0.42	0.0 ^b^	0.0 ^b^
Tricosylic acid	C23:0	0.0 ^b^		12.32 ± 0.07 ^a^	12.25 ± 0.44 ^a^
Lignoceric acid	C24:0	0.0 ^c^		17.78 ± 0.15 ^b^	19.73 ± 0.20 ^a^
Palmitoleic acid	C16:1	0.0 ^c^		13.64 ± 0.14 ^b^	20.30 ± 0.30 ^a^
Oleic acid	C18:1	22.3 ± 0.9 ^a^	26.9	12.42 ± 0.43 ^c^	19.85 ± 0.12 ^b^
Linoleic acid	C18:2	40.0 ± 2.3 ^a^	54.3	15.49 ± 0.06 ^b^	13.64 ± 0.14 ^c^
Linolenic acid	C18:3	6.0 ± 1.1	0.99	0.0	0.0
SFA		12.7	11.13	41.45	46.18
UFA		68.3	82.19	41.55	53.79

Means designated with a different letter in the same row are significantly different (*p* ≤ 0.05). SFA: saturated fatty acids; UFA: unsaturated fatty acids. The *p* values were recorded *p* = 0.0000 *** for the fatty acid contents of the three protein meals. The fatty acid spectrum of corn oil was derived from the literature.

**Table 5:** Trace element contents detected in soybean, *B. zonata* and *S. littoralis* meals in %.

Elements (%)	Soybean Meal	*B. zonata* Meal	*S. littoralis* Meal
Calcium (Ca)	1.03 ± 0.02 ^c^	4.79 ± 0.11 ^a^	3.42 ± 0.08 ^b^
Potassium (K)	2.24 ± 0.05 ^c^	4.67 ± 0.12 ^b^	6.26 ± 0.13 ^a^
Iron (Fe)	1.92 ± 0.02 ^b^	0.87 ± 0.02 ^c^	1.31 ± 0.01 ^a^
Zinc (Zn)	3.12 ± 0.05 ^a^	2.6 ± 0.05 ^b^	1.5 ± 0.02 ^c^
Magnesium (Mg)	1.2 ± 0.03 ^c^	9.15 ± 0.12 ^a^	2.81 ± 0.03 ^b^
Phosphorous (P)	0.41 ± 0.01 ^c^	8.80 ± 0.14 ^a^	2.3 ± 0.05 ^b^
Sulphur (S)	0.0 ^c^	0.94 ± 0.04 ^a^	0.67 ± 0.01 ^b^
Chlorine (Cl)	0.0 ^c^	0.84 ± 0.02 ^a^	0.78 ± 0.02 ^b^

Means designated with the different letters in the same row are significantly different (*p* ≤ 0.05). The *p* values were recorded as *p* = 0.0000 *** for all trace element contents.

**Table 6:** Average live body weight, feed intake and relative weight (%) of carcass and selected organs in Japanese quails along the trial, depending on the diet.

Feed Performance (Mean ± SE)	Soybean Meal	*B. zonata* Meal	*S. littoralis* Meal	MSE	*p*-Value
Initial weight (g) (at day 7)	22.7 ± 0.5	22.1 ± 0.4	22.5 ± 0.5	0.222	0.46
Final weight (g) (38 days; at day 45)	195.1 ± 1.4 ^c^	208.5 ± 1.5 ^a^	203.1 ± 1.5 ^b^	0.389	0.0 ***
Body weight gain (g/day) (7–45 days)	4.5 ± 0.5	4.9 ± 0.4	4.7 ± 0.8	0.033	0.094
Feed intake (g/day) (7–45 days)	10.1 ± 0.7 ^a^	9.0 ± 0.3 ^b^	9.3 ± 0.5 ^b^	0.051	0.003
Feed conversion ratio (feed intake: body weight gain)	2.2 ± 0.02 ^a^	1.8 ± 0.01 ^b^	2.0 ± 0.02 ^b^	0.014	0.011
Relative weight (%) of carcass and selected organs (±SE)					
Carcass	68.5 ± 0.3 ^b^	73.1 ± 0.2 ^a^	72.8 ± 0.3 ^a^	0.047	0.0 ***
Liver	2.67 ± 0.4	2.93 ± 0.01	2.86 ± 0.04	0.003	0.0028
Heart	0.69 ± 0.51	0.71 ± 0.05	0.70 ± 0.03	1.67	0.244
Spleen	0.1 ± 0.01	0.15 ± 0.01	0.14 ± 0.01	7.78	0.007

Means designated with different letters in the same row are significantly different (*p* ≤ 0.05). SE: standard error of the three replicates, respectively; MSE: mean squared error.

**Table 7:** Blood profile and hormonal level of Japanese quails at 45 days of age after feeding trials with three different diets.

Parameters	Soybean Meal	*B. zonata* Meal	*S. littoralis* Meal	MSE	*p* Value
Total protein (g/dL)	3.4 ± 0.05	3.8 ± 0.03	3.7 ± 0.5	0.002	0.0 ***
Albumin (g/dL)	1.65 ± 0.01 ^a^	1.42 ± 0.02 ^b^	1.37 ± 0.01 ^c^	1.0	0.0 ***
Globulin (g/dL)	1.75 ± 0.02 ^a^	1.65 ± 0.03 ^b^	1.50 ± 0.01 ^c^	3.2	0.0 ***
Albumin:globulin ratio	1.1 ± 0.01 ^b^	0.86 ± 0.01^a^	0.91 ± 0.02 ^a^	0.024	0.0046
Total antioxidants (mM/L)	1.6 ± 0.01	1.4 ± 0.01	1.1 ± 0.01	0.041	0.196
ALP (IU/L)	84.4 ± 2.2	86.4 ± 3.2	82.111.9	5.157	0.1527
ALT (U/mL)	38.7 ± 1.1 ^a^	37.4 ± 0.7 ^a^	34.63 ± 0.9 ^b^	0.777	0.0038
AST (U/mL)	33.8 ± 0.5	34.0 ± 0.8	33.70 ± 0.4	0.218	0.6815
Triglyceride (mg/dL)	178.7 ± 2.3 ^b^	189.8 ± 3.2 ^a^	193.4 ± 2.5 ^a^	6.358	0.0009
Cholesterol (mg/dL)	190.3 ± 3.2 ^a^	200.5 ± 2.1 ^b^	209.1 ± 2.9 ^c^	6.533	0.0003

Means designated with different letters in the same row are significantly different (*p* ≤ 0.05). SE: standard error of the respective three replicates, respectively; MSE: mean squared error; ALP: alkaline phosphates activity; ALT: alanine aminotransferase activity; AST: aspartate aminotransferase activity.

## References

[1] Mungkung R., Aubin J., Prihadi T.H., Slembrouck J., van der Werf H.M.G., Legendre M. (2013). Life Cycle Assessment for environmentally sustainable aquaculture management: A case study of combined aquaculture systems for carp and tilapia. J. Clean. Prod..

[2] Beski S.S.M., Swick R.A., Iji P.A. (2015). Specialized protein products in broiler chicken nutrition: A review. Anim. Nutr..

[3] Womeni H.M., Tiencheu B., Linder M., Nabayo E.M., Tenyang N., Mbiapo T.F., Villeneuve P., Fanni J., Parmentier M. (2012). Nutritional Value and Effect of Cooking, Drying and Storage Process on Some Functional Properties of Rhynchophorus Phoenicis. Int. J. Life Sci. Pharma. Res..

[4] Bovera F., Loponte R., Marono S., Piccolo G., Parisi G., Iaconisi V., Gasco L., Nizza A. (2016). Use of larvae meal as protein source in broiler diet: Effect on growth performance, nutrient digestibility, and carcass and meat traits. J. Anim. Sci..

[5] Van Huis A., van Itterbeeck J., Klunder H., Mertens E., Halloran A., Muir G., Vantomme P. (2013). Edible Insects. Future Prospects for Food and Feed Security.

[6] Zetina D.H., Correa M.d.S.C., García-Figueroa J., Pino J.M., Ramos-Elorduy J., Neto E.M.C. (2007). Knowledge about useful entomofauna in the county of La Purisima Palmar de Bravo, Puebla State, Mexico. Biotemas.

[7] Veldkamp T., van Duinkerken G., Van Huis A., Lakemond C.M.M., Ottevanger E., Bosch G., Van Boekel M. (2012). Insects as a Sustainable Feed Ingredient in Pig and Poultry Diets—A Feasibility Study.

[8] Ravi C., Jeyashree A., Devi K.R. (2011). Antimicrobial Peptides from Insects: An Overview. RIB.

[9] Barroso F.G., de Haro C., Sánchez-Muros M.-J., Venegas E., Martínez-Sánchez A., Pérez-Bañón C. (2014). The potential of various insect species for use as food for fish. Aquaculture.

[10] Belforti M., Gai F., Lussiana C., Renna M., Malfatto V., Rotolo L., de Marco M., Dabbou S., Schiavone A., Zoccarato I. (2016). Tenebrio Molitor Meal in Rainbow Trout (Oncorhynchus Mykiss) Diets: Effects on Animal Performance, Nutrient Digestibility and Chemical Composition of Fillets. Ital. J. Anim. Sci.

[11] Gasco L., Henry M., Piccolo G., Marono S., Gai F., Renna M., Lussiana C., Antonopoulou E., Mola P., Chatzifotis S. (2016). Tenebrio molitor meal in diets for European sea bass (*Dicentrarchus labrax* L.) juveniles: Growth performance, whole body composition and in vivo apparent digestibility. Anim. Feed Sci. Technol..

[12] Renna M., Schiavone A., Gai F., Dabbou S., Lussiana C., Malfatto V., Prearo M., Capucchio M.T., Biasato I., Biasibetti E. (2017). Evaluation of the suitability of a partially defatted black soldier fly (*Hermetia illucens* L.) larvae meal as ingredient for rainbow trout (*Oncorhynchus mykiss* Walbaum) diets. J. Anim. Sci. Biotechnol..

[13] Belghit I., Liland N.S., Waagbø R., Biancarosa I., Pelusio N., Li Y., Krogdahl Å., Lock E.-J. (2018). Potential of insect-based diets for Atlantic salmon (*Salmo salar*). Aquaculture.

[14] Bukkens S.G.F. (1997). The nutritional value of edible insects. Ecol. Food. Nutr..

[15] Schiavone A., Cullere M., de Marco M., Meneguz M., Biasato I., Bergagna S., Dezzutto D., Gai F., Dabbou S., Gasco L. (2016). Partial or total replacement of soybean oil by black soldier fly larvae (*Hermetia illucens* L.) fat in broiler diets: Effect on growth performances, feed-choice, blood traits, carcass characteristics and meat quality. Ital. J. Anim. Sci.

[16] Pomalégni S.C.B., Gbemavo D.S.J.C., Kpadé C.P., Kenis M., Mensah G.A. (2017). Traditional use of fly larvae by small poultry farmers in Benin. J. Insects Food Feed.

[17] Dutton R., Komblas K.N., Green M.B., Lyon D.J.B.d. (1989). Twenty years of use of chlorpyrifos in cotton in Egypt. Pest Management in Cotton: Symposium.

[18] Kenis M., Hien K., Vantomme P., Münke C., van Huis A., van Itterbeek J., Hakman A. (2014). Prospects and constraints for the use of insects as human food and animal feed in West Africa. Proceedings of the Book of Abstracts, International Conference Insects to Feed the World.

[19] Makkar H.P.S., Tran G., Heuzé V., Ankers P. (2014). State-of-the-art on use of insects as animal feed. Anim. Feed Sci. Technol..

[20] Diener S., Zurbrügg C., Tockner K. (2015). Bioaccumulation of heavy metals in the black soldier fly, Hermetia illucens and effects on its life cycle. J. Insects Food Feed.

[21] Basiouny A., Ghoneim K., Tanani M., Hamadah K., Waheeb H. (2016). Disturbed protein content in Egyptian cotton leafworm Spodoptera littoralis (Boisd.) (Lepidoptera: Noctuidae) by some novel chitin synthesis inhibitors. Int. J. Adv. Res. Biol. Sci..

[22] Danieli P.P., Ronchi B., Speranza S. (2016). Alternative animal protein sources for aquaculture: A preliminary study on nutritional traits of Mediterranean brocade (*Spodoptera littoralis*, Boisduval) larvae. Ital. J. Anim. Sci..

[23] Alikwe P.C.N., Faremi A.Y., Egwaikhide P.A. (2010). Biochemical Evaluation of Serum Metabolites, Enzymes and Haematological Indices of Broiler-Chicks Fed with Varying Levels of Rumen Epithelial Scraps in Place of Fish Meal Proteins. Res. J. Poult. Sci..

[24] Albalasmeh A.A., Berhe A.A., Ghezzehei T.A. (2013). A new method for rapid determination of carbohydrate and total carbon concentrations using UV spectrophotometry. Carbohydr. Polym..

[25] Folch J., LEES M., Sloane Stanley G.H. (1957). A simple method for the isolation and purification of total lipides from animal tissues. J. Biol. Chem..

[26] AOAC (2002). Official Methods of Analysis of the Association of Official Analytical Chemists.

[27] Knapp D.R. (1979). Handbook of Analytical Derivatization Reactions.

[28] NRC (1994). Nutrient Requirements of Poultry.

[29] Soares R.d.T., Fonseca J.B., Santos A.d.O.d., Mercandante M.B. (2003). Protein requirement of Japanese quail (Coturnix coturnix japonica) during rearing and laying periods. Rev. Bras. Cienc. Avic..

[30] Duncan D.B. (1955). Multiple Range and Multiple F Tests. Biometrics.

[31] Rumpold B.A., Schlüter O.K. (2013). Nutritional composition and safety aspects of edible insects. Mol. Nutr. Food Res..

[32] Al-Qazzaz M.F.A., Ismail D., Akit H., Idris L.H. (2016). Effect of using insect larvae meal as a complete protein source on quality and productivity characteristics of laying hens. R. Bras. Zootec..

[33] Józefiak D., Józefiak A., Kierończyk B., Rawski M., Świątkiewicz S., Długosz J., Engberg R.M. (2016). 1. Insects—A Natural Nutrient Source for Poultry—A Review. Ann. Anim. Sci..

[34] Bukkens S.G.F., Paoletti M.G. (2005). Insects in the human diet: nutritional aspects. Ecological Implications of Minilivestock: Potential of Insects, Rodents, Frogs, and Snails.

[35] Bednářová M. (2013). Possibilities of Using Insects as Food in the Czech Republic (Dissertation).

[36] Khosravi H., Mehri M., Bagherzadeh-Kasmani F., Asghari-Moghadam M. (2016). Methionine requirement of growing Japanese quails. Anim. Feed Sci. Technol..

[37] DeFoliart G.R. (1992). Insects as human food. Crop Prot..

[38] Akullo J., Agea J.G., Obaa B.B., Okwee-Acai J., Nakimbugwe D. (2018). Nutrient composition of commonly consumed edible insects in the Lango sub-region of northern Uganda. Int. Food Res. J..

[39] Bhulaidok S., Sihamala O., Shen L., Li D. (2010). Nutritional and fatty acid profiles of sun-dried edible black ants (*Polyrhachis vicina* Roger). Maejo Int. J. Sci Technol..

[40] Ramos-Elorduy J., Moreno J.M.P., Prado E.E., Perez M.A., Otero J.L., de Guevara O.L. (1997). Nutritional Value of Edible Insects from the State of Oaxaca, Mexico. J. Food Compos. Anal..

[41] Phillips J., Burkholder W. (1995). Allergies related to food insect production and consumption. Food Insects Newsl..

[42] Finke M.D. (2002). Complete nutrient composition of commercially raised invertebrates used as food for insectivores. Zoo Biol..

[43] Sirimungkararat S., Saksirirat W., Nopparat T., Natongkham A., Durst P.B. (2010). Edible products from eri silkworm (*Samia ricini* D.) and mulberry silkworm (*Bombyx mori* L.) in Thailand. Forest Insects as Food: Humans Bite Back: Proceedings of a Workshop on Asia-Pacific Resources and Their Potential for Development, 19–21 February 2008, Chiang Mai, Thailand.

[44] Raksakantong P., Meeso N., Kubola J., Siriamornpun S. (2010). Fatty acids and proximate composition of eight Thai edible terricolous insects. Food Res. Int..

[45] Ekpo K.E., Onigbinde A.O., Asia I.O. (2009). Pharmaceutical potentials of the oils of some popular insects consumed in southern Nigeria. Afr. J. Pharm. Pharmacol..

[46] DTU Food Frida fooddata.dk: Food ID: 1415 (Corn oil). https://frida.fooddata.dk/food/1415?lang=en.

[47] North American Menopause Society (2001). The role of calcium in peri- and postmenopausal women: Consensus opinion of The North American Menopause Society. Menopause.

[48] Oonincx D.G.A.B., van Itterbeeck J., Heetkamp M.J.W., van den Brand H., van Loon J.J.A., van Huis A. (2010). An exploration on greenhouse gas and ammonia production by insect species suitable for animal or human consumption. PLoS ONE.

[49] Hyun S.-H., Kwon K.H., Park K.-H., Jeong H.C., Kwon O., Tindwa H., Han Y.S. (2012). Evaluation of nutritional status of an edible grasshopper, Oxya Chinensis Formosana. Entomol. Res..

[50] Gao Q., Wang X., Wang W., Lei C., Zhu F. (2017). Influences of chromium and cadmium on the development of black soldier fly larvae. Environ. Sci. Pollut. Res. Int..

[51] Van der Fels-Klerx H.J., Camenzuli L., van der Lee M.K., Oonincx D.G.A.B. (2016). Uptake of Cadmium, Lead and Arsenic by Tenebrio molitor and Hermetia illucens from Contaminated Substrates. PLoS ONE.

[52] Van Huis A. (2013). Potential of insects as food and feed in assuring food security. Annu. Rev. Entomol..

[53] Muzzarelli R.A.A. (2010). Chitins and chitosans as immunoadjuvants and non-allergenic drug carriers. Mar. Drugs.

[54] Lee K.P., Simpson S.J., Wilson K. (2008). Dietary protein-quality influences melanization and immune function in an insect. Funct. Ecol..

[55] Khoushab F., Yamabhai M. (2010). Chitin research revisited. Mar. Drugs.

[56] Young D.H., Köhle H., Kauss H. (1982). Effect of Chitosan on Membrane Permeability of Suspension-Cultured Glycine max and Phaseolus vulgaris Cells. Plant Physiol..

[57] El Ghaouth A., Arul J., Asselin A., Benhamou N. (1992). Antifungal activity of chitosan on post-harvest pathogens: Induction of morphological and cytological alterations in Rhizopus stolonifer. Mycol. Res..

[58] Elangovan A.V., Mandal A.B., Tyagi P.K., Tyagi P.K., Toppo S., Johri T.S. (2004). Effects of enzymes in diets with varying energy levels on growth and egg production performance of Japanese quail. J. Sci. Food Agric..

[59] Kaur S., Mandal A.B., Singh K.B., Kadam M.M. (2008). The response of Japanese quails (heavy body weight line) to dietary energy levels and graded essential amino acid levels on growth performance and immuno-competence. Livest. Sci..

[60] Gheisari A., Halaji H.A., Maghsoudinegad G., Toghyani M., Alibemani A., Saeid S.E. (2011). Effect of Different Dietary Levels of Energy and Protein on Performance of Japanese Quails (*Coturnix coturnix* Japonica). 2nd International Conference on Agricultural and Animal Science. IPCBEE.

[61] Donaldson J., Madziva M.T., Erlwanger K.H. (2017). The effects of high-fat diets composed of different animal and vegetable fat sources on the health status and tissue lipid profiles of male Japanese quail (*Coturnix coturnix* japonica). Asian-Australas J. Anim. Sci..

[62] Liu D., Veit H.P., Wilson J.H., Denbow D.M. (2003). Long-term supplementation of various dietary lipids alters bone mineral content, mechanical properties and histological characteristics of Japanese quail. Poult. Sci..

[63] Oyegoke O.O., Akintola A.J., Fasoranti F.O. (2006). Dietary potentials of the edible larvae of Cirina forda (westwood) as a poultry feed. Afr. J. Biotechnol..

[64] Widjastuti T., Wiradimadja R., Rusmana D. (2014). The effect of substitution of fish meal by Black Soldier Fly (*Hermetia illucens*) maggot meal in the diet on production performance of quail (*Coturnix coturnix* japonica). Anim. Sci..

[65] Wang X., Gao Q., Liu X., Wang X.-P., Lei C., Sayed W.A.A., Zhu F. (2018). Metallothionein in Hermetia illucens (Linnaeus, 1758) larvae (Diptera: Stratiomyidae), a potential biomarker for organic waste system. Environ. Sci. Pollut. Res. Int..

[66] Adeniji A. (2007). Effect of Replacing Groundnut Cake with Maggot Meal in the Diet of Broilers. Int. J. Poult. Sci..

[67] Ijaiya A.T., Eko E.O. (2009). Effect of Replacing Dietary Fish Meal with Silkworm (*Anaphe infracta*) Caterpillar Meal on Performance, Carcass Characteristics and Haematological Parameters of Finishing Broiler Chicken. Pakistan J. Nutr..

[68] Loponte R., Nizza S., Bovera F., de Riu N., Fliegerova K., Lombardi P., Vassalotti G., Mastellone V., Nizza A., Moniello G. (2017). Growth performance, blood profiles and carcass traits of Barbary partridge (*Alectoris barbara*) fed two different insect larvae meals (*Tenebrio molitor* and *Hermetia illucens*). Res. Vet. Sci..

[69] Marono S., Loponte R., Lombardi P., Vassalotti G., Pero M.E., Russo F., Gasco L., Parisi G., Piccolo G., Nizza S. (2017). Productive performance and blood profiles of laying hens fed Hermetia illucens larvae meal as total replacement of soybean meal from 24 to 45 weeks of age. Poult. Sci..

[70] Bovera F., Piccolo G., Gasco L., Marono S., Loponte R., Vassalotti G., Mastellone V., Lombardi P., Attia Y.A., Nizza A. (2015). Yellow mealworm larvae (*Tenebrio molitor*, L.) as a possible alternative to soybean meal in broiler diets. Br. Poult. Sci..

[71] Duwa H., Saleh B., Igwebuike J.U. (2014). The replacement of fish meal with maggot meal on the performance, carcass characteristic, hematological and serum biochemical indices of growing rabbits. Glob. J. Bio-Sci. Biotechnol..

